# Bilateral simultaneous hip arthroplasty shows comparable early outcome and complication rate as staged bilateral hip arthroplasty for patients scored ASA 1-3 if performed by a high-volume surgeon

**DOI:** 10.1007/s00264-023-05871-1

**Published:** 2023-06-24

**Authors:** Stephanie Kirschbaum, Robert Hube, Carsten Perka, Christophe Ley, Simone Rosaria, Michael Najfeld

**Affiliations:** 1 Centre for Musculoskeletal Surgery, Charité-University Hospital Berlin, Charitéplatz 1, 10117 Berlin, Germany; 2 OCM Orthopädische Chirurgie München, Steinerstraße 6, 81369 Munich, Germany; 3 University of Luxembourg, 2 Av. de l’Universite, 4365 Esch-sur-Alzette, Luxembourg; 4 University of Napoli Federico II, Corso Umberto I 40, 80138 Naples, Italy

**Keywords:** Bilateral simultaneous hip arthroplasty, One-stage hip arthroplasty, Two-stage hip arthroplasty, Staged hip arthroplasty, Complications

## Abstract

**Purpose:**

The aim of this study was to compare early outcomes after simultaneous and staged hip arthroplasty (THA) in patients with bilateral symptomatic pathology.

**Methods:**

We conducted a retrospective cohort study including all patients scheduled for primary THA for bilateral hip osteoarthritis (OA, *n* = 290). Patients either received simultaneous (*n* = 152, 52.4%) or staged (*n* = 138, 47.6%) bilateral THA based on individual preference. All operations (*n* = 428) were performed by one single, high-volume surgeon. Demographic data (e.g., age, ASA score) as well as perioperative parameters (haemoglobin drop (Hb), red blood cell transfusion, length of stay (LOS), operation time, six week complication rate and achievement of inpatient rehabilitation key points) were evaluated.

**Results:**

Patients in the simultaneous bilateral THA group were younger (62.8 ± 8.9 vs. 65 ± 9.7 years, *p* = 0.022) and had lower ASA scores (1.8, (34.2% ASA 1, 55.3% ASA 2, 37.2% ASA 3) vs. 2.0 (18.8% ASA 1, 61.6% ASA 2, 19.6% ASA 3)) than the staged group. While the average LOS was 7.1 ± 1.7 days for simultaneous bilateral THA, the combined LOS for the staged group was 12.9 ± 2.4 days (*p* < 0.001). The cumulative operation time in the simultaneous bilateral THA group was 61.1 ± 11.5 min and 57.6 ± 7.3 min in the staged group (*p* < 0.015). Cumulative Hb loss was significantly higher in the staged group (2.1 ± 7.2 g/dl vs. 3.7 ± 1.3 g/dl, *p* < 0.001). No significant differences were found concerning the complication rate or early inpatient rehabilitation.

**Conclusion:**

Simultaneous bilateral hip arthroplasty in patients with symptomatic bilateral hip osteoarthritis is as safe and successful as a staged procedure if performed by a high-volume surgeon.

## Introduction

To date, there is no consensus regarding the optimal surgical strategy for patients presenting with severe bilateral hip pain and OA at their first consultation. While staged bilateral THA represents the gold standard, patients regularly ask for simultaneous bilateral THA, as it provides some organizational comfort: one anaesthesia, one hospital stay, one rehabilitation period [1], one period of impaired mobility and therefore only one loss of independence. Furthermore, one-stage bilateral THA results in lower costs for the health care system, up to 27% [2–4].

Nevertheless, surgeons hesitate to perform simultaneous bilateral THA, and only 0.9–1.1% of all THAs are performed as simultaneous bilateral THA [5, 6]. This restraint may be due to the reported higher transfusion rate and higher risk of infection or deep-vein thrombosis (DVT) [7–11]. However, other authors found no significant differences concerning thromboembolic events, cardiovascular complications, blood loss or transfusion rate [2, 11, 12]. Unfortunately, most of the studies only included patients with American Society of Anesthesiologists (ASA) scores of I or II [13–17] or lacked any evaluation of early or mid-term functional outcomes [1, 2, 8, 14, 18].

To our knowledge, there is only one single-centre study comparing simultaneous and staged bilateral THA performed by a single, high-volume arthroplasty surgeon. As that study lacks any information about the ASA score, exclusion criteria and functional outcomes, no general conclusion concerning risks and safety is possible. Therefore, the aim of this study was to compare surgical and medical complications, as well as blood loss and early functional outcomes, associated with simultaneous bilateral THA compared to staged bilateral THA performed by a single, high-volume arthroplasty surgeon.

## Patients and methods

The local Research Ethics Committee has confirmed that no ethical approval is required (confirmation number: 21004). In this retrospective, single-centre cohort study, only patients with simultaneous or staged (within 12 months) bilateral THA procedures for symptomatic bilateral osteoarthritis of the hip operated by the same high-volume surgeon between January 2015 and December 2020 were included (*n* = 366). Every patient was offered either a simultaneous or staged procedure explaining known advantages and disadvantages as described in the literature [7–9, 11]. As this shared decision-making determined the choice of a simultaneous or staged procedure, there was no further randomization or matching of the groups. Exclusion criteria were a time frame >12 months between staged THA (*n* = 76) or previous surgeries (trauma, osteotomy) making one-stage implant removal immediately before THA necessary as those would have impacted operation time and blood loss (*n* = 0). Patients with posttraumatic osteoarthritis or other deformities (Perthes disease, former osteotomy) without any implant in situ were still included. No patient was excluded due to ASA scoring.

**Fig. 1:**
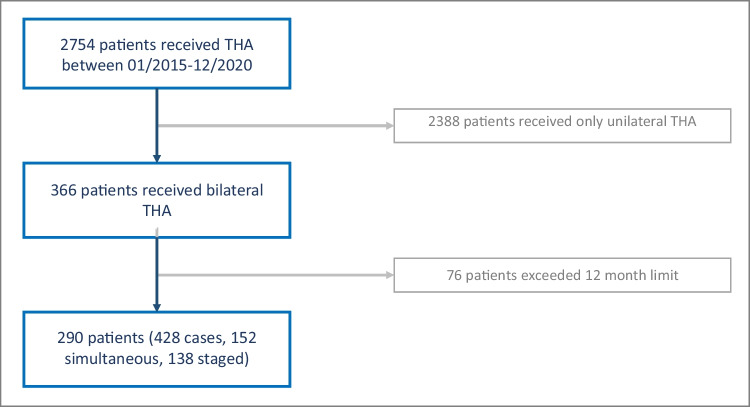
Flowchart demonstrating patients’ enrolment

In total, *n* = 290 patients (*n* = 428 cases) were included in the study. The patients were divided into two groups: one-stage (*n* = 152, 52.4%) versus staged THA (*n* = 138, 47.6% (Fig. 1)).

### THA protocol

All surgeries were performed using a muscle sparing anterolateral approach for THA and cementless stems (Fitmore stem Fa. Zimmer or SL-MIA stem Fa. Smith and Nephew). All departments had implemented standard protocols (1-g tranexamic acid intravenous and 3-g intraarticular, opioid-sparing analgesia). The postoperative rehabilitation regimens were the same for the simultaneous or staged procedures: physiotherapy started the day of surgery with full weight-bearing using crutches or walker.

### Demographic characteristics and outcome parameters

Patient records were assessed for patient-related and demographic measures, including age (years), sex, body mass index (BMI kg/m^2^) and American Society of Anesthesiologists (ASA) score. Furthermore, postoperative haemoglobin drop (Hb), need for red blood cell transfusion, length of stay (LOS) and operation time were assessed. The haemoglobin drop was analysed by comparison of the preoperative blood haemoglobin level (g/dl) to the blood haemoglobin level the day after surgery. The length of stay started with inpatient treatment the day before surgery when surgical and anaesthesiologic elucidation of the patient is performed and ended with patient discharge. Discharge criteria were a dry wound, unaccompanied walk in the ward and passed the stair assessment. The time needed to reach the mentioned rehabilitation key points (unaccompanied walk and assessment of stairs under control of our physiotherapists) was also evaluated. Operation time was defined between the incision of the skin and finishing the wound closure.

Medical (e.g., symptomatic deep vein thrombosis or thromboembolism) and surgical (e.g., hematoma, wound healing disorders, infection, fracture) complications within 6 weeks after surgery were documented as well. Every patient received a follow-up 6 weeks after surgery at the institutional outpatient department for the identification of early complications and radiographic assessment.

### Radiographic outcome

Every patient received scaled, standardized radiographic assessments (pelvic anterior-posterior in the standing position) before the first surgery, at discharge and six weeks after (second) THA using the pelvis anteroposterior view. The leg length discrepancy and occurring subsidence of the stem were evaluated on radiographs taken 6 weeks after THA. In case of staged bilateral THA, leg length discrepancy was evaluated six weeks after second THA. An independent investigator (MN), blinded to the study group, measured the absolute difference between the upper margin of the minor trochanter and a horizontal line between the acetabular tear drop figures (mm). The incidence of significant leg length differences after bilateral THA (>5 mm) was compared between the simultaneous and staged groups.

### Statistical analysis

Statistical analysis was performed using SPSS Version 26 (SPSS Inc., Chicago, IL, USA). Data are expressed as the mean ± standard deviation and range. The Shapiro–Wilk test was used to test the Gaussian distribution. Next, comparison of the parametric data was performed with the Mann–Whitney test or *t*-test. The chi-square test was used to compare the distribution of ordinal variables. The Kruskal–Wallis test and ANOVA were used to identify differences in metric parameters between patients with ASA scores 1–3.

Correlations between the metric and ordinal parameters were calculated via the Kendall tau-b test, and correlations between the metric parameters were calculated with a Pearson correlation test. The significance level of all tests was 5% (2-sided). In case *t*-test was used, 95% confidence interval was additionally reported.

## Results

A total of 290 patients (202 men (47.2%), 226 women (52.8%)) with 428 cases were included in the study. The average age at the time of surgery was 64.2 ± 9.4 (32–87) years, and the average BMI was 25.6 ± 4.1 (18–40) kg/m^2^. The overall ASA score was 1.9 ± 0.6 (1–3). Nobody was scored ASA 4. A total of 152 patients underwent simultaneous bilateral THA, and 138 patients underwent staged bilateral THA (276 cases). The average time between THAs in the staged procedure was 226 ± 101 (43–265) days.

There were significant differences in the demographic parameters between the simultaneous and staged groups (Table 1).

**Table 1:** Comparison of demographic data between the simultaneous and staged bilateral THA groups

	Simultaneous bilateral THA (*n* = 152)	Staged bilateral THA (*n* = 276)	Significance
Age (year)	62.8 ± 8.9 (32–81)	65.0 ± 9.7 (37–87)	0.022
Sex (female/male, %)	46.1/53.9	56.5/43.5	0.079
BMI (kg/m^2^)	25.7 ± 4.0 (18–40)	25.6 ± 4.2 (18–40)	0.553
Average ASA score	1.8 ± 0.6 (1–3)	2 ± 0.6 (1–3)	0.004
	34.2% ASA 1	18.8% ASA 1	
	55.3% ASA 2	61.6% ASA 2	
	37.2% ASA 3	19.6% ASA 3	

*ASA* American Society of Anesthesiologists, *BMI* body mass index, *THA* total hip arthroplasty

### Outcome parameters

Simultaneous procedures took longer than isolated and cumulative staged procedure (*p* < 0.012) while cumulative Hb loss was significantly higher in the staged group (Table 2). The transfusion rate for both groups was 0%. Post hoc power analysis of cumulative Hb drop as well as cumulative operation time showed a power of 99.9% (effect size cumulative Hb drop = 0.696, effect size cumulative operation time = 0.762).

**Table 2:** Comparison of key points concerning perioperative parameters and inpatient mobilization between simultaneous and staged groups

	Simultaneous bilateral THA	Staged bilateral THA	Significance
Surgical parameters			
Operation time (min)	61.1 ± 11.5 (19.5–95.5)	28.8 ± 4.6 (20–60)	<0.001
Cumulative operation time (min)		57.6 ± 7.3 (44–86)	0.012
Hb drop per surgery (g/dl)	2.1 ± 7.2 (0.2–4.3)	1.9 ± 0.7 (1.9–0.7)	0.004
Cumulative Hb drop (g/dl)		3.7 ± 1.3 (1.1–10.7)	<0.001
Rehabilitation key points			
Average LOS (days)	7.1 ± 1.7 (4–18)	6.5 ± 1.5 (3–15)	<0.001
Cumulative LOS (days)		12.9 ± 2.4 (6–21)	<0.001
Free walking at ward (at day)	2.2 ± 0.9 (1–5)	2.3 ± 0.8 (1–5)	0.265
Exercising stair climbing (at day)	3.8 ± 1.1 (2–7)	3.6 ± 1 (1–7)	0.614

*THA* total hip arthroplasty, *Hb* haemoglobin, *LOS* length of stay

The average LOS was significantly longer for simultaneous procedure (Table 2). Post hoc power analysis of cumulative LOS also showed a power of 99.9% (effect size cumulative LOS = 0.819).

No significant difference was found between simultaneous and staged bilateral THAs concerning the key points of early inpatient rehabilitation (Table 2).

### Complications

The overall complication rate was 2.3% (*n* = 10). Whereas 1.4% (*n* = 6, 4× wound revision due to severe haematoma, 1× gastritis, 1× ileus) of these complications occurred after simultaneous bilateral THA, 0.9% (*n* = 4, 2× periprosthetic fracture Vancouver type B2, 1× gastritis, 1× acute coronary syndrome) occurred after staged bilateral THA (*p* = 0.177). No deaths or symptomatic thromboembolic events occurred within six weeks after surgery for any of the patients.

### Radiographic outcomes

There was no significant difference concerning the incidence of leg length discrepancies between the groups (*p* = 0.324). No subsidence was noted.

### Influence of ASA score on surgical and functional outcomes

Independent of the simultaneous or staged procedure, there were no significant differences in LOS, Hb drop per surgery or the achievement of key rehabilitation marks between patients who scored ASA 1, 2 or 3 (Table 3).

**Table 3:** Comparison of key points concerning perioperative parameters and inpatient mobilization depending on ASA scoring

	ASA score 1	ASA score 2	ASA score 3	Significance
Age (years)	63.6 ± 8.9 (32–81)	65.3 ± 9.2 (41–87)	61.4 ± 10.6 (37–81)	0.028
BMI (kg/m^2^)	23.2 ± 2.6 (19–31)	25.3 ± 3.0 (18–37)	31.4 ± 4.4 (23–40)	<0.001
LOS (days)	6.9 ± 1.9 (4–18)	6.6 ± 1.5 (3–15)	6.7 ± 1.7 (4–12)	0.574
Cumulative operation time (min)	67.6 ± 11.7 (46–96)	67.1 ± 14.0 (44–111)	69.4 ± 15.3 (35–105)	0.534
Hb drop per surgery (g/dl)	2.0 ± 0.7 (0.9–4.6)	1.9 ± 0.7 (0.2–3.9)	2.1 ± 1 (0.7–6.7)	0.604
Cumulative Hb drop (g/dl)	2.6 ± 1.2 (0.9–7.9)	2.9 ± 1.1 (0.2–7.4)	3.4 ± 1.9 (0.7–10.7)	0.02
Free walking at ward (at day)	2.3 ± 0.9 (1–5)	2.2 ± 0.8 (1–5)	2.5 ± 0.8 (1–5)	0.088
Exercising stair climbing (at day)	3.7 ± 1 (1–7)	3.8 ± 1 (2–7)	3.8 ± 1.2 (2–7)	0.931

*ASA* American Society of Anesthesiologists, *BMI* body mass index, *Hb* haemoglobin, *LOS*, length of stay

Independent of the simultaneous or staged procedure, the ASA score only showed a significant correlation with BMI (Kendall tau-b 0.460, *p* < 0.001) and cumulative Hb drop (Kendall tau-b 0.133, *p* = 0.004).

Comparing only those patients scored ASA 3, there were no significant differences concerning outcome parameters between staged and simultaneous groups. Higher incidence of complications in simultaneous group showed no significance. The same could be demonstrated for patients scored ASA 1. Patients scored ASA 2 showed significant shorter LOS and faster assessment of stairs and less Hb drop per surgery (Table 4).

**Table 4:** Comparison of key points concerning perioperative parameters and inpatient mobilization between simultaneous and staged groups depending on ASA scoring

	Simultaneous bilateral THA	Staged bilateral THA	Significance
ASA score 1 (*n* = 104)			
Average operation time (min)	57.9 ± 8.9 (43–81)	27.9 ± 4.1 (22–40)	<0.001
Cumulative operation time (min)	57.9 ± 8.9 (43–81)	55.9 ± 7.0 (46–76)	0.300
Hb drop per surgery (g/dl)	2.0 ± 0.6 (0.9–4.0)	1.9 ± 0.7 (0.9–4.6)	0.613
LOS (days)	7.2 ± 2.2 (4–18)	6.6 ± 1.4 (4–10)	0.072
Free walking at ward (at day)	2.1 ± 0.9 (1–5)	2.5 ± 0.8 (1–4)	0.008
Exercising stair climbing (at day)	3.7 ± 1.1 (3–7)	3.7 ± 0.9 (1–5)	0.690
Complications (%) of all ASA 1	5.8	1.9	0.309
ASA score 2 (*n* = 254)			
Average operation time (min)	62.3 ± 11.1 (47–96)	28.3 ± 4.3 (20–60)	<0.001
Cumulative operation time (min)	62.3 ± 11.1 (47–96)	56.6 ± 6.6 (44–86)	<0.001
Hb drop per surgery (g/dl)	2.0 ± 0.7 (0.2–4.1)	1.8 ± 0.6 (0.5–4.1)	<0.001
LOS (days)	7.1 ± 1.4 (4–10)	6.4 ± 1.5 (3–15)	<0.001
Free walking at ward (at day)	2.3 ± 0.9 (1–5)	2.2 ± 0.8 (1–4)	0.469
Exercising stair climbing (at day)	3.9 ± 1.1 (2–7)	3.6 ± 1.0 (2–7)	0.022
Complications (%) of all ASA 2	2.4	0.6	0.255
ASA score 3 (*n* = 70)			
Average operation time (min)	65.8 ± 17.5 (20–90)	31.1 ± 5.5 (23–47)	<0.001
Cumulative operation time (min)	65.8 ± 17.5 (20–90)	62.3 ± 7.9 (48–80)	0.370
Hb drop per surgery (g/dl)	2 ± 0.9 (0.7–4.3)	2.1 ± 1.0 (0.7–6.7)	0.763
LOS (days)	7 ± 1.9 (5–12)	6.7 ± 1.6 (4–11)	0.738
Free walking at ward (at day)	2.4 ± 0.7 (1–3)	2.5 ± 0.8 (1–5)	0.645
Exercising stair climbing (at day)	3.8 ± 1.0 (3–6)	3.7 ± 1.1 (2–7)	0.408
Complications (%) of all ASA 3	6.3	3.7	0.547

*ASA* American Society of Anesthesiologists, *Hb* haemoglobin, *LOS* length of stay

## Discussion

Since Jaffe and Charnley first reported simultaneous bilateral THA in 1971 [19], many studies have evaluated the controversial risks and benefits of simultaneous bilateral THA compared with staged bilateral THA. The small number of patients included, the existing selection bias (including only ASA score 1 or 2) and the missing evaluations of the functional outcomes limit the conclusions of most existing studies. Therefore, the recent study is the first to demonstrate equal results concerning early rehabilitation outcomes and the six week complication rate of simultaneous bilateral THA performed by a single high-volume surgeon independent from ASA scoring.

There were no major differences between simultaneous and staged bilateral THAs concerning the perioperative transfusion rate, early functional outcome or complication rate. It is very interesting that cumulative blood loss of staged procedure is higher than blood loss of simultaneous THA of both hips. This observation might be explained by the surgeon’s increased awareness of bleeding management concerning the longer operation time in simultaneous procedure. This increased awareness might also be the reason for equal blood loss of patients scored ASA 3 between simultaneous and staged THA procedures if operated in an adequate setting. However, the reduced blood loss in the simultaneous group represents a major advantage, as postoperative anaemia has been shown to inhibit postoperative rehabilitation [20–22]. In addition to postoperative anaemia, the persisting pain in the contralateral (unaddressed) hip inhibits recovery. Yoshii et al. described that the range of motion and activity of daily living were significantly greater in patients treated with simultaneous bilateral THA than in patients with staged bilateral THA for bilateral hip osteoarthritis [23]. Subsequently, the authors concluded that recovery from bilateral hip osteoarthritis could not be achieved until both hips received THA. This is a very interesting observation, as simultaneous bilateral THA is usually shown to cause more intense pain after surgery, which is also discussed as a risk factor for delayed recovery [24]. On the other hand, the recent study was not able to find any differences concerning early inpatient recovery directly postoperatively when pain is assumed to be most intense. This disadvantage of simultaneous procedure therefore appears to be manageable with sufficient analgesia.

The successful early rehabilitation in both groups might also represent a protective factor against thromboembolic events [25] and could serve as an explanation for the missing symptomatic thromboembolic events in the recent study. In contrast to older data, no higher incidence of thromboembolic or cardiovascular events was found in simultaneous bilateral THA [10, 26]. However, this fact might be less attributed to the surgical procedure of simultaneous vs. staged THA and more to the improved perioperative and surgical standards of modern THA. This might explain why other recent studies were also not able to find significant differences in DVT or thromboembolic events between simultaneous and staged THAs [4, 12, 27, 28].

One recent meta-analysis by Huang et al. demonstrated that simultaneous bilateral THA is associated with a higher risk (OR 2.17) of surgical site infection than staged procedures [28]. The authors thought that different study designs, surgical techniques and perioperative blood management were possible explanations, as the complication rates did not differ. However, when comparing LOS, rehabilitation duration, outcome and complication rates of simultaneous and staged bilateral THA, the cumulative data of staged procedure should always be compared to those of simultaneous procedure. Otherwise, the authors are not respecting the fact that patients who undergo staged bilateral THAs experience two times the risk of a single procedure.

While more healthy patients were more likely to have simultaneous procedures, there were interestingly no differences concerning LOS, transfusion rate or rehabilitation outcome. Only BMI and cumulative Hb drop were correlated with higher ASA scores. Furthermore, ASA 3 patients—so those with the highest risk factors—demonstrated no worse outcome between staged and simultaneous procedures. These data might encourage surgeons to not only offer a one-stage procedure to young and healthy patients but also to ASA 3 score patients, as no differences in outcome but an increased cumulative blood loss were found. Therefore, patients who score ASA 3 also seemed to benefit from the simultaneous procedure.

Recent study confirmed findings of longer cumulative LOS in staged bilateral THA [4, 12] and was also able to demonstrate that simultaneous bilateral THA only increases LOS by one day compared to a single procedure of staged bilateral THA. This is an important finding, as the combination of reduced LOS in simultaneous procedures as well as single operating and anaesthetic sessions reduces costs for the health care system by approximately 30% [3, 29, 30]. Furthermore, a simultaneous procedure is not associated with an increased rehabilitation period, which means an additional advantage regarding the cost reduction for the health care system: only one in- and one outpatient rehabilitation must be paid instead of two, saving between 1111 and 2047 € (=1157–2133 USD) per case [31]. Additionally, the simultaneous procedure significantly reduces the time of impairment for the patients’ daily lives, as patients must organize their family life, family or friend support and rehabilitation appointments only once, leading to a faster recovery of autonomy.

The recent study has several limitations. First, the cohorts were not matched for age, BMI or ASA score due to the retrospective study design. This might lead to a selection bias, as the simultaneous group showed a slightly better ASA score and younger age. However, unlike existing studies [13–17], no exclusion was made due to age, ASA score exceeding grade 2 or complexity of osteoarthritis (e.g. dysplasia, post-traumatic). The range of the operation time implicates that no patient was excluded due to complexity of THA procedure. Furthermore, the decision of simultaneous or staged procedure was based on the patients’ informed decision—not on ASA scoring or age. Additionally, a detailed analysis concerning influence of ASA scoring on overall outcome as well as separated outcome was performed to overcome the lack of matching. Another limitation is that the occurrence of complications was only evaluated within 6 weeks after surgery, which might cause the authors to miss later complications, such as surgical site or periprosthetic joint infections, periprosthetic fracture or subsidence of femoral stem. However, complications like subsidence of the femoral stem literature reported highest incidence within the first 6 weeks after surgery [32–34]. It can therefore be assumed that most of the sintering as well as intraoperative occult fractures would have been identified during the six week follow-up. As most surgery-associated complications, such as DVT or thromboembolism, occur within the early rehabilitation period (first 7 days [35]), most of those complications should have been assessed too, as average LOS and therefore clinical control in this study was 7 days. Another limitation might represent the fact that postoperative Hb drop is measured the day after surgery and was not regularly controlled in further follow-up even if some literature demonstrated major Hb drop occurs at day three or four after surgery [36]. However – this limitation impacts simultaneous and staged groups equally and furthermore, there was no need for any transfusion so the possible bias seems negligible. A final limitation is the strict use of a cementless femoral stem component in all patients. There are registry data recommending THA with a cemented femoral stem in patients over 75 years of age [37, 38] to reduce revision rates, while other studies discuss controversially the increased mortality due to a higher incidence of thromboembolic events when using cemented femoral stems [39, 40]. Therefore, the current study is reluctant to extend its conclusion to patients over 75 years of age receiving cemented HTEP.

In contrast, a strength of the present study is certainly the monocentric, single-surgeon design with highly standardised procedures which excludes a bias with regard to surgical procedure and rehabilitation.

## Conclusion

Simultaneous bilateral THA represents a safe procedure in patients with bilateral symptomatic hip OA that is not associated with a significant higher incidence of red blood cell transfusion or complications as symptomatic DVT, intraoperative fracture or femoral stem subsidence. If performed by a high-volume surgeon in an adequate setting, also patients scored ASA 3 are safe for a simultaneous procedure which offers reduced costs for the health care system as well as a reduced LOS with equal early rehabilitation, which favours patient regain of autonomy.

## Data Availability

Not applicable for general access. Of course, we can provide original data if wished by the editors.

## Abbreviations

ASA: American Society of Anaesthesiology
BMI: body mass index
DVT: deep venous thrombosis
Hb: haemoglobin
LOS: length of stay
THA: total hip arthroplasty
